# Olfactory and gustatory functioning and food preferences of patients with Alzheimer’s disease and mild cognitive impairment compared to controls: the NUDAD project

**DOI:** 10.1007/s00415-019-09561-0

**Published:** 2019-10-08

**Authors:** A. S. Doorduijn, M. A. E. de van der Schueren, O. van de Rest, F. A. de Leeuw, J. L. P. Fieldhouse, M. I. Kester, C. E. Teunissen, P. Scheltens, W. M. van der Flier, M. Visser, S. Boesveldt

**Affiliations:** 1 Department of Nutrition and Dietetics, Amsterdam UMC, Vrije Universiteit Amsterdam, Amsterdam Public Health Research Institute, PO Box 7057, 1007 MB Amsterdam, The Netherlands; 2 Alzheimer Center Amsterdam, Department of Neurology, Amsterdam Neuroscience, Vrije Universiteit Amsterdam, Amsterdam UMC, Amsterdam, The Netherlands; 3 Department of Nutrition and Health, HAN University of Applied Sciences, Nijmegen, The Netherlands; 4 Division of Human Nutrition and Health, Wageningen University & Research, Wageningen, The Netherlands; 5 Neurochemistry Laboratory, Department of Clinical Chemistry, Amsterdam Neuroscience, Vrije Universiteit Amsterdam, Amsterdam UMC, Amsterdam, The Netherlands; 6 Department of Health Sciences, Faculty of Science, Vrije Universiteit Amsterdam and The Amsterdam Public Health Research Institute, Amsterdam, The Netherlands

**Keywords:** Older adults, Malnutrition, Dementia, Cognition

## Abstract

Our aim is to compare olfactory and gustatory function and food preferences of patients with Mild Cognitive Impairment (MCI) and Alzheimer’s disease (AD) with controls. We included 22 patients with MCI, 30 patients with AD and 40 controls and assessed olfactory threshold, odor discrimination and odor identification (Sniffin’ Sticks), gustatory functioning (Taste Strips), and food preferences (Macronutrient and Taste Preference Ranking Task). Linear regression analyses were used to study associations of five cognitive domains or AD biomarkers with olfactory functioning. Groups did not differ in olfactory threshold, gustatory function and food preferences. Patients with MCI and AD scored lower on odor discrimination and identification than controls. Poorer memory, but no other cognitive domain, was associated with poorer odor discrimination and odor identification, but not with odor threshold. No associations with AD biomarkers were found. In conclusion, patients with MCI and AD have poorer odor discrimination and identification ability than controls, but similar detection thresholds. This is likely a consequence of poorer memory rather than directly caused by AD pathology.

**Electronic supplementary material:**

The online version of this article (10.1007/s00415-019-09561-0) contains supplementary material, which is available to authorized users.

## Introduction

Unintended weight loss and malnutrition are common features in patients with Mild Cognitive Impairment (MCI) and Alzheimer’s disease (AD) dementia, with prevalence rates ranging from 0–60% [1-4]. It is unknown whether this weight loss is due to a higher energy expenditure, reduced dietary intake, a combination of both, or that it is influenced by other factors. Dietary intake is partly driven by olfactory and gustatory functioning and food preferences [5]. Detecting and identifying a smell or taste plays an important role in liking of food products, but also has a functional role (e.g., to signal spoiled food or nutrient content) [5]. With aging, a decrease in olfactory and gustatory function may change food preferences and thereby dietary patterns [6-10]. Furthermore, poor olfactory function is associated with higher mortality in older adults [11].

Olfactory and gustatory function, and to lesser extent food preferences, have been studied in patients with AD, but rarely in patients with MCI. Poorer olfactory function, and especially poorer odor identification, have been reported in patients with MCI and AD, while results on threshold detection and ability to discriminate odors are inconsistent [12, 13]. Some authors also found that specific olfactory tests, like the peanut butter odor detection test, might be useful in early detection of AD, although more research is needed [14].

Gustatory function was assessed by only a few studies in patients with AD, and results are conflicting. Some studies found a lower overall sense of taste in patients with AD compared to controls [15-17] while others did not [18]. Furthermore, only one study included patients with MCI, showing similar gustatory function as patients with AD [16]. In addition, food preferences for specific taste or macronutrient categories have rarely been studied in patients with AD and never in patients with MCI. It is reported that patients with AD consume more energy from carbohydrate-rich products compared to age-matched controls, which might be caused by a higher preference for sweet products [19, 20]. We will compare olfactory threshold, discrimination and identification, gustatory function, taste intensity preference and food preferences of patients with MCI and AD with cognitively normal controls. It is currently not clear whether poorer olfactory and gustatory function and altered food preferences are a consequence of poorer cognitive performance or caused by AD pathology [21]. Therefore, we will study associations of cognitive performance on different domains or AD biomarkers in cerebrospinal fluid (CSF) for parameters that differ between diagnosis groups.

## Methods

### Participants

The NUDAD (Nutrition, the Unrecognized Determinant in Alzheimer’s Disease) study is a prospective cohort studying nutritional determinants in AD and pre-dementia stages, with 3 year clinical follow-up. The total NUDAD cohort (*n* = 552) includes all patients of the Amsterdam Dementia Cohort, who visited our Alzheimer center between September 2015 and August 2017, were diagnosed with AD dementia, MCI or subjective cognitive decline (SCD) and had a Mini-Mental State Examination (MMSE) score > 16 [22]. All patients underwent a standardized dementia screening, including extensive neuropsychological assessment, neurological examination and laboratory tests [23]. MCI and probable AD were diagnosed according to the corresponding National Institute on Aging-Alzheimer’s Association criteria [24, 25]. As controls, we used individuals with SCD who presented with memory complaints at our memory clinic, but performed normal on all clinical examinations (i.e., criteria for MCI, dementia or any other neurological or psychiatric disorder that could explain their cognitive complaints were not met) [26].

Of the NUDAD participants, a subgroup participated in an in-depth study on nutrition and related factors, including measurements of smell, taste and food preferences. The subgroup consisted of 92 participants, 30 patients with AD dementia, 22 patients with MCI and 40 controls. For this subgroup, inclusion criteria were age ≥ 50 years, MMSE score ≥ 19, medically stable (assessed by physician) and a sufficient knowledge of the Dutch language. Exclusion criteria were current smoking, having major psychiatric disorder, neurological disorders known to influence smell and taste other than AD, severe food allergy, severe diseases of the digestive tract, or recent diagnosis of cancer other than basal cell carcinoma of the skin. Informed consent was obtained from all participants and the local Medical Ethical Committee approved the study.

Descriptive characteristics included: age, gender, MMSE score, measured body mass index (BMI, in kg/m^2^), level of education and vegetarian diet (yes or no). Level of education was assessed using the Verhage classification system [27], which we categorized into low (score 1–3), intermediate (score 4 and 5) and high (score 6 and 7).

### Olfactory and gustatory functioning

Subjective olfactory changes were evaluated by asking participants if they experienced a change in their sense of smell in the past months (yes or no). If yes, they rated their general ability on each olfactory domain (detecting, discriminating, and identifying odors) on a 5-point scale, ranging from ‘not at all’ to ‘very good’ [28]. Olfactory testing consisted of three parts: detection threshold level (T), discrimination ability (D) and odor identification (I), and was measured using Sniffin’ Sticks (Burghart, Wedel, Germany) [29]. Detection threshold test consisted of 16 triplets, within each triplet 2 pens had no odorant and one pen contained a dilution of *n*-butanol. The pens were presented in random order, participants were blindfolded and had to identify the odorant, starting with the lowest concentration. If the odorant was successfully identified in two successive trials, lower concentrations were offered until the participant did not identify the odorant anymore, and then a higher concentration was provided. The threshold level was defined as the mean of the final 4 out of 7 staircase reversals, ranging from 1 to 16. For discrimination ability, 16 triplets were presented, each triplet contained two pens with the same odor and one different. The pens within a triplet were offered in randomized order and the blindfolded participants had to discriminate which pen smelled different. The identification test consisted of 16 pens containing common and well-known odors at supra-threshold level. With each pen, a form with four odor names was presented. Participants had to identify the correct odor in this forced multiple-choice setting. Each correct discrimination and identification resulted in 1 point, with the sum score ranging from 0 to 16. Overall olfactory function (TDI) score was the sum of the three tests ranging from 1 to 48 [29, 30]. Participant’s olfactory function was classified as anosmia (TDI ≤ 15.9), hyposmia (TDI 16–30.3) and normosmia (TDI ≥ 30.3) [30]*.*

Gustatory functioning was evaluated using Taste Strips (Burghart, Wedel, Germany), comprising of 16 filter paper strips impregnated with four basic tastes (sweet, salt, sour, bitter) in four different concentrations [31]. Strips were presented in block-randomized order, starting with the lowest concentration. Taste strips were placed on the tongue, participants closed their mouth and were forced to make a choice for each strip between sweet, salt, sour, bitter or tasteless. Participants rinsed their mouth with water before each taste strip. Each correct answer resulted in 1 point, ranging from 0 to 4 for each basic taste and 0 to 16 for the total score.

### Taste intensity preferences

Preference for taste intensities was measured by having the participants taste lemonade and tomato juice, with five different concentrations of sugar and salt, respectively. Sugar concentrations ranged from 0.0625 mol/l (M) to 1 M, salt from 0.03125 M to 0.5 M, with a twofold increase between every concentration. The middle concentrations represent sugar and salt concentrations in commercially available beverages. Samples of 25 ml were randomly presented, and participants had to taste the samples with a sip of water in between. In the ranking part, they had to rank the samples from least (score of 1) to most liked (score of 5). For the liking part, performed after the food preference task, they had to rate each sample on a 100-point visual analogue scale (VAS), anchored ‘do not like at all’ to ‘like extremely’.

### Food preference

Food preferences were assessed using the Macronutrient and Taste Preference Ranking Task (MTPRT [32]). This is a validated computer-based tool presenting pictures of food products from four macronutrient categories (high protein, high carbohydrate, high fat and low energy), including both sweet and savory products. All food products are well known and commercially available in the Netherlands. The task was performed about one and a half hour after breakfast. Liking of all 32 products was assessed on a 100-point VAS, anchored ‘do not like at all’ to ‘like extremely’ and averaged per category. In the ranking task, participants had to rank four pictures according to ‘what they most desire to eat at this moment’, each picture was presented two times in different trials. In the macronutrient section, each picture represented one macronutrient category. The preference score per macronutrient was calculated and ranged from 1 to 4, with a higher score indicating a higher preference. In the taste section, each trial consisted of two pictures from two macronutrient categories, one sweet and one savory. The high protein category was excluded as it only contains savory products. The preference score for taste was calculated and ranged from 1.5 to 3.5. The preference score for sweet and savory is each other’s opposite, therefore only scores for sweet are reported in this article.

Olfactory discrimination and identification, gustatory functioning, taste intensity preferences and the MTPRT were executed using EyeQuestion software (Logic8 BV).

### Neuropsychological assessment

Cognitive performance was assessed using a standardized neuropsychological test battery covering five domains; memory [(visual association test (VAT); rey auditory verbal learning task [33, 34]], attention [Trail Making Test part A (TMT A); digit span forward; Stroop test, word and color subtasks [35-37]], executive functioning (frontal assessment battery; digit span backward; Stroop test color-word subtask; letter fluency [36-39]), language (category fluency [animal naming]; the naming condition of the VAT [33, 40]) and visuospatial ability (dot counting; fragmented letters; number location [41]). Raw test scores were converted into z-scores using the mean and SD of our study population. Test scores for TMT A were log-transformed because they were not normally distributed. Z-scores for TMT A and Stroop were inverted, such that lower scores indicate worse cognitive performance. Domain scores were calculated by averaging z-scores of the individual tests within that domain, if at least two tests were available.

### AD biomarkers

Cerebrospinal fluid (CSF) was obtained by lumbar puncture using a 25-gauge needle, and collected in 10 ml polypropylene tubes (Sarstedt) following standardized protocols [42]. β-Amyloid 42 (Aβ_42_), tau and phosphorylated tau (p-tau) levels were determined with sandwich Innotest ELISAs (Fujirebio, Ghent, Belgium) [43] and available of 57 participants (62%). Aβ_42_ levels were adjusted for the drift that occurred over the years [44].

### Statistical analysis

Between-diagnosis group differences in participant characteristics, olfactory and gustatory functioning, taste intensity preferences and food preferences were tested using ANOVA with post-hoc Bonferroni adjusted *t* tests for normally distributed continuous variables, and Chi-square tests for categorical variables. ANOVAs of olfactory and gustatory functioning, taste intensity preferences and food preferences were adjusted for age, gender and education. Some meat and fish products are included in the MTPRT (mainly as part of the high protein category), therefore adherence to a vegetarian diet was added as a possible confounder when analyzing the liking and preference scores. Within the total cohort, we used linear regression analyses to evaluate associations of cognitive domains or AD biomarkers in CSF (independent variables) with olfactory functioning (dependent variables). All descriptive variables in Table 1 that changed the regression coefficient ≥ 10% were considered to be a possible confounder and included in the model. The associations of cognitive domains with olfactory functioning were adjusted for age, gender and education. Similarly, the linear regression analyses of AD biomarkers with olfactory functioning were adjusted for age and gender. To test the assumptions of the regression analyses, we plotted and checked residuals of all models, which were all normally distributed. Significance was set at *p* < 0.05. All analyses were performed with SPSS version 22 (released 2013, IBM SPSS Statistics for Windows, Armonk, NY, USA).

**Table 1:** Characteristics of the NUDAD study population according to diagnosis group

	Controls		MCI		AD dementia		*P* value across groups
	*N*		*N*		*N*		
Age (years)	40	62.5 ± 6.8	22	69.8 ± 7.2^†^	30	69.5 ± 9.4^†^	**< 0.001**
Gender, female	40	22 (55.0)	22	6 (27.3)	30	16 (53.3)	0.770
MMSE score	40	29 [27–30]	22	26 [25–28]^†^	30	24 [21-26]^†,‡^	**< 0.001**
BMI (kg/m^2^)	40	25.6 ± 6.3	22	25.1 ± 3.3	30	26.3 ± 4.9	0.595
Level of education	40		22		30		**0.008**
Low		1 (2.5)		1 (4.5)		2 (6.7)^†^^,‡^	
Intermediate		12 (30.0)		6 (27.3)		18 (60.0)^†^^,‡^	
High		27 (67.5)		15 (68.2)		10 (33.3)^†^^,‡^	
Vegetarian diet	40	7 (17.5)	22	1 (4.5)	30	4 (13.3)	0.349
*AD biomarkers in CSF*							
Aβ_42_ (pg/ml)	25	1023 ± 222	15	728 ± 234^†^	18	615 ± 223^†^	**< 0.001**
Tau (pg/ml)	24	340 ± 182	15	403 ± 267	18	696 ± 330^†^^,‡^	**0.004**
P-Tau (pg/ml)	24	48 ± 35	15	58 ± 30	18	85 ± 32^†^^,‡^	**0.002**
*Cognitive domain scores*							
Memory	40	0.93 ± 0.58	20	− 0.19 ± 0.48^†^	29	− 0.81 ± 0.52^†^^,‡^	**< 0.001**
Attention	40	0.38 ± 0.52	20	− 0.13 ± 0.68	29	− 0.53 ± 1.14^†^	**< 0.001**
Executive functioning	38	0.44 ± 0.56	19	− 0.24 ± 0.63^†^	21	− 0.61 ± 0.73^†^	**< 0.001**
Language	40	0.47 ± 0.50	20	− 0.19 ± 0.74^†^	28	− 0.56 ± 1.00^†^	**< 0.001**
Visuospatial ability	34	0.23 ± 0.28	17	0.11 ± 0.49	19	− 0.51 ± 1.31^†^^,‡^	**0.004**

Data in mean ± SD; *n* (%); median [interquartile range]. Normally distributed continuous variables were tested using ANOVA with post-hoc Bonferroni adjusted *t* tests, and Chi-square tests for categorical variables

*AD* Alzheimer’s disease, *MCI* Mild Cognitive Impairment, *MMSE* Mini-Mental State Examination, *BMI* body mass index, *CSF* cerebrospinal fluid

^†^Significantly different from controls upon post-hoc testing;

^‡^Significantly different from MCI upon post-hoc testing

## Results

Patients with MCI and AD dementia were older and had a lower MMSE score compared to controls, and patients with AD dementia were lower educated than patients with MCI and controls (Table 1). Diagnosis groups did not differ in BMI. Patients with MCI and AD dementia had lower Aβ_42_ levels and higher tau and p-tau levels than controls. Performance on all cognitive domains differed between groups, with controls scoring highest and patients with AD dementia scoring lowest.

**Fig. 1:**
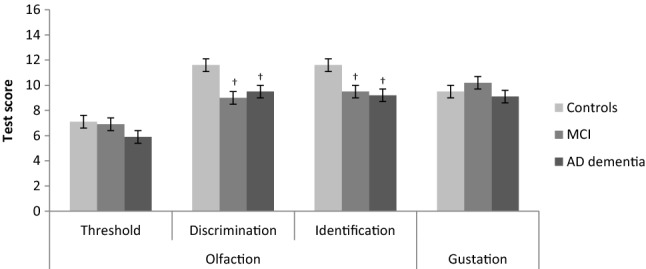
Mean olfactory (threshold, discrimination, and identification) and gustatory functioning scores according to diagnosis group. Age, gender, and education-adjusted ANOVA with post-hoc Bonferroni adjusted *t* tests were used. Error-bars represent SE. *AD* Alzheimer’s disease, *MCI* mild cognitive impairment. †Significantly different from controls upon post-hoc testing

None of the patients with AD dementia, four patients with MCI (18%) and five controls (13%) reported a subjective change in olfactory functioning over the past months (Table 2). On testing, patients with MCI and AD dementia had a lower TDI score and were more often anosmic or hyposmic than controls (Table 2). Specifically, patients with MCI and AD dementia scored lower on odor discrimination and identification compared to controls (9.0 and 9.5 vs. 11.6; 9.5 and 9.2 vs. 11.6, respectively) (Fig. 1). Groups did not differ in olfactory detection threshold. Patients with AD dementia scored lower on taste sour compared to patients with MCI, but total taste scores did not differ between diagnosis groups.

**Table 2:** Olfactory (dys)function and gustatory functioning per basic taste according to diagnosis group

	Controls	MCI	AD dementia	*P* value across groups
Subjectively reported change in olfactory function past months	5 (12.5)	4 (18.2)	0 (0.0)^†^^,^^‡^	0.069
TDI score	30.2 ± 1.1	25.5 ± 1.4^†^	24.6 ± 1.3^†^	**0.004**
Classification olfactory (dys)function				**0.004**
Anosmia	0 (0)	3 (13.6)^†^	5 (16.7)^†^	
Hyposmia	15 (37.5)	12 (54.5)^†^	18 (48.9)^†^	
Normosmia	25 (62.5)	7 (31.8)^†^	7 (23.3)^†^	
Gustatory functioning				
Sweet	3.0 ± 0.2	3.3 ± 0.2	2.9 ± 0.2	0.426
Salt	2.2 ± 0.2	2.4 ± 0.3	2.5 ± 0.2	0.572
Sour	2.2 ± 0.1	2.5 ± 0.2	1.7 ± 0.2^‡^	**0.022**
Bitter	2.0 ± 0.2	2.0 ± 0.3	1.9 ± 0.2	0.938

Data in mean ± SE or n (%). Age, gender and education adjusted ANOVA with post-hoc Bonferroni-adjusted *t* tests were used for continuous variables and Chi-square tests for categorical variables

*AD* Alzheimer’s disease, *MCI* mild cognitive impairment, *TDI* sum of threshold, discrimination and identification

^†^Significantly different from controls upon post-hoc testing

^‡^Significantly different from MCI upon post-hoc testing

Adjusted linear regression analyses showed that poorer performance on the cognitive domain memory was associated with lower TDI, odor discrimination and odor identification scores, but not with threshold detection (Table 3). There were no associations for the four other cognitive domain scores. Moreover, no associations between AD biomarker levels and olfactory functioning were observed (Table 4).

**Table 3:** Associations of cognitive domains with olfactory functioning

	TDI	Threshold	Discrimination	Identification
Memory	**3.10 (1.51; 4.69)**	0.61 (− 0.16; 1.38)	**1.19 (0.57; 1.82)**	**1.29 (0.60; 2.01)**
Attention	− 0.17 (− 1.72; 1.37)	− 0.41 (− 1.09; 0.26)	0.14 (− 0.46; 0.74)	0.10 (− 0.60; 0.80)
Executive functioning	0.64 (− 1.86; 3.14)	− 0.42 (− 1.58; 0.74)	0.20 (− 0.78; 1.18)	0.86 (− 0.21; 1.92)
Language	0.54 (− 1.68; 2.76)	− 0.32 (− 1.31; 0.67)	0.31 (− 0.57; 1.19)	0.55 (− 0.45; 1.55)
Visuospatial ability	− 0.91 (− 2.76; 0.94)	− 0.17 (− 1.01; 0.67)	− 0.50 (− 1.22; 0.22)	− 0.24 (− 1.02; 0.53)

Data presented as β (95% CI) (regression coefficients and 95% confidence interval). Age, gender and education-adjusted linear regression analyses with cognitive domains (independent variables) and olfactory functioning (dependent variables)

*TDI* sum of threshold, discrimination and identification

**Table 4:** Associations of AD biomarkers in CSF with olfactory functioning

	TDI	Threshold	Discrimination	Identification
Aβ_42_	0.00 (− 0.00; 0.01)	0.00 (− 0.00; 0.00)	0.00 (0.00; 0.01)	0.00 (− 0.00; 0.00)
Tau	0.00 (− 0.01; 0.01)	0.00 (− 0.00; 0.00)	0.00 (− 0.00; 0.00)	− 0.00 (− 0.00; 0.00)
P-Tau	− 0.01 (− 0.07; 0.04)	0.01 (− 0.02; 0.03)	− 0.01 (− 0.03; 0.01)	− 0.01 (− 0.04; 0.01)

Data presented as β (95% CI) (regression coefficients and 95% confidence interval). Age and gender adjusted linear regression analyses with AD biomarkers (independent variables) and olfactory functioning (dependent variables)

*AD* Alzheimer’s disease, *CSF* cerebrospinal fluid, *Aβ*_*42*_ β-amyloid 42, *p-tau* phosphorylated tau, *TDI* sum of threshold, discrimination and identification

**Fig. 2:**
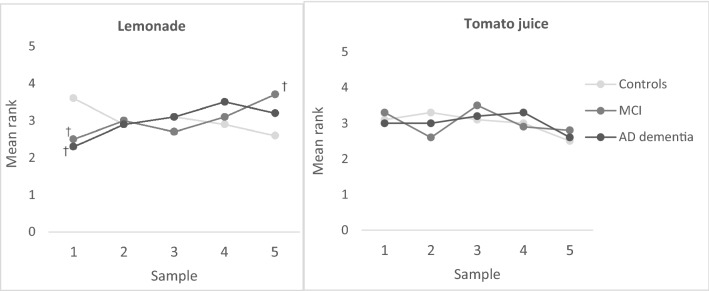
Mean ranking of taste intensity preferences lemonade and tomato juice according to diagnosis group. Tested using age, gender, and education-adjusted ANOVA with post-hoc Bonferroni adjusted *t* tests. *AD* Alzheimer’s disease, *MCI* Mild Cognitive Impairment; sample 1 represents the lowest concentration of sugar or salt, sample 5 the highest concentration. †Significantly different from controls upon post-hoc testing

For the taste intensity preferences, patients with MCI and AD dementia ranked the least sweet lemonade lower than controls (Fig. 2), while patients with MCI ranked the sweetest lemonade higher than controls, i.e., overall, controls tended to prefer lower concentrations of sugar in the lemonade. Similarly, evaluating the liking scores, patients with MCI and AD dementia rated the sweetest lemonade higher than controls (Supplementary Table A). Groups did not differ in ranking and liking scores for tomato juice.

The results of the MTPRT showed that groups did not differ in liking scores and preference scores of any taste or macronutrient category (Table 5). Adherence to a vegetarian diet (*n* = 12) did not affect group differences in liking and preference scores (data not shown).

**Table 5:** Liking and preference scores of macronutrient and taste categories of the macronutrient and taste preference ranking task per diagnosis group

	Controls	MCI	AD dementia	*P* value across groups
*Liking scores*				
Sweet	60.1 ± 2.4	64.6 ± 3.1	67.7 ± 2.8	0.145
Savory	57.2 ± 2.0	55.9 ± 2.7	54.9 ± 2.3	0.790
High protein	62.9 ± 3.2	60.1 ± 4.2	54.3 ± 3.7	0.257
High carbohydrate	46.9 ± 2.7	47.8 ± 3.5	53.3 ± 3.1	0.306
High fat	55.6 ± 2.6	60.2 ± 3.5	64.1 ± 3.1	0.148
Low energy	67.7 ± 2.5	68.6 ± 3.3	67.2 ± 2.9	0.947
*Preference scores*				
Sweet	2.7 ± 0.1	2.7 ± 0.1	2.8 ± 0.1	0.696
High protein	2.7 ± 0.1	2.5 ± 0.1	2.4 ± 0.1	0.326
High carbohydrate	2.0 ± 0.1	2.1 ± 0.1	2.3 ± 0.1	0.174
High fat	2.4 ± 0.1	2.6 ± 0.1	2.6 ± 0.1	0.364
Low energy	2.9 ± 0.1	2.8 ± 0.1	2.8 ± 0.1	0.736

Data in Mean ± SE. Tested using age, gender and education adjusted ANOVA with post-hoc Bonferroni adjusted *t* tests

*AD* Alzheimer’s disease, *MCI* Mild Cognitive Impairment

As we observed no differences between diagnosis groups in gustatory function, taste intensity preferences and food preferences, we did not further explore associations between cognitive domains or AD biomarkers and these parameters.

## Discussion

The main finding of this study is that patients with MCI and AD dementia scored lower on odor discrimination and identification compared to controls, but groups did not differ in olfactory threshold, gustatory function or food preferences. Poorer memory scores were associated with lower odor discrimination and identification scores.

Compared to normative data [30], controls scored normal on all olfactory tests according to their age and gender. In our sample patients with MCI and AD dementia were more likely to suffer from hyposmia (impaired olfactory functioning) or anosmia (no sense of smell) than controls. This seems conflicting with the subjective olfactory complaints reported by controls and patients with MCI, although it is well known that discrepancies between self-reported and results of olfactory tests are present [45]. In line with literature [16, 46] our study suggests that patients with MCI and AD dementia do not have a lower sensitivity for detecting odors but do have difficulties in discriminating and identifying odors. Odor discrimination and identification are complex cognitive tasks because olfactory perception needs to be compared with information stored in memory and labelled verbally, whereas a detection threshold is less dependent of cognitive performance [47-49]. This is further supported by the association of the cognitive domain memory with both odor discrimination and identification, but not with threshold detection. Furthermore, we did not find associations of any other cognitive domain nor of the AD biomarkers with olfactory functioning. Thus, our results suggest that poorer memory, rather than AD pathology or decline on other cognitive domains, might explain the poorer odor discrimination and identification in patients with MCI or AD dementia.

Overall gustatory function did not differ across diagnosis groups and all scored within the normal range for their age [50]. This is in contrast to another study that reported lower scores on all basic tastes and total gustatory function in patients with MCI and AD dementia compared to controls [16]. Discrepancies might be due to the larger age differences across groups in our study, or our adjustment (for age, gender and education) of the analysis.

The MTPRT was uniquely applied to patients with MCI and AD dementia. We did not observe any difficulties in performing the task and all food products were recognized by the participants. The diagnosis groups did not differ in liking and preference scores for any taste or macronutrient category, while patients with MCI and AD dementia did have a higher preference for more intense sweet tastes in the taste intensity task. This seems conflicting, but taste intensity preference is determined by one specific factor within a product (i.e., sugar or salt concentration), while food preferences are measured across different food products and categories. Our study suggests that, while the sense of (sweet) taste and food preferences seem unaltered, patients with MCI and AD dementia do prefer a more intense sweet taste.

Among the strengths of our study is the use of standardized olfactory and gustatory functioning, taste intensity and food preferences tests. Furthermore we had both neuropsychological tests in five different domains and multiple AD biomarkers in CSF available. This study also has some limitations. First, because of the cross-sectional design no causal inferences can be made. Future research should focus on olfactory and gustatory functioning in a longitudinal design and should explore how changes in cognitive performance over time are associated with changes in olfactory and gustatory functioning. We are currently following our participants with annual neuropsychological assessment and will re-evaluate odor identification and gustatory function after 2 years. A second limitation is that our population is small, relatively young, and in early stage of the disease based on a MMSE score of 19 or higher and living independent, which makes it hard to generalize the results of our study. It might be that alterations in gustatory function or food preferences appear in a more advanced stage of the disease. Furthermore, our MCI group consisted of both patients with amnestic as well as non-amnestic MCI. Although we excluded participants with a major psychiatric disorder, we could not exclude that lower level psychiatric disorders might influenced our results. A final limitation is that the AD biomarkers were only available in 62% of our sample. Although the subgroup with biomarkers available seemed representative for the total study sample (mean ± SD TDI participants with AD biomarkers 27.0 ± 8.2, without AD biomarkers 27.4 ± 7.1, *p* = 0.795), the subgroup is small which limits the power of detecting associations. Furthermore, future studies should investigate the associations of atrophy degree of hippocampus and entorhinal cortex in relation to olfactory function and memory.

In conclusion, patients with MCI and AD dementia had more difficulties in discriminating and identifying odors compared to controls, while no differences in threshold detection were observed. Poorer score on the cognitive domain memory was associated with lower odor discrimination and identification scores while no associations with other domains were observed. Furthermore, AD biomarkers were not associated with olfactory functioning, suggesting that AD pathology itself is not related to olfactory function. No differences between diagnosis groups were observed for overall gustatory function and food preferences, although patients with MCI and AD dementia had a higher preference for more intense sweet tastes. Further research is needed to study the specific role of odor discrimination and identification in food choice and dietary pattern in patients with different stages of cognitive decline.

## Electronic supplementary material

Below is the link to the electronic supplementary material.

Supplementary file1 (DOCX 13 kb)

## References

[1] Droogsma E (2013). Nutritional status of community-dwelling elderly with newly diagnosed Alzheimer's disease: prevalence of malnutrition and the relation of various factors to nutritional status. J Nutr Health Aging.

[2] Gillioz AS (2009). Spared and impaired abilities in community-dwelling patients entering the severe stage of Alzheimer's disease. Dement Geriatr Cogn Disord.

[3] Galesi LF, Leandro-Merhi VA, de Oliveira MR (2013). Association between indicators of dementia and nutritional status in institutionalised older people. Int J Older People Nurs.

[4] Malara A (2014). Relationship between cognitive impairment and nutritional assessment on functional status in Calabrian long-term-care. Clin Interv Aging.

[5] McCrickerd K, Forde CG (2016). Sensory influences on food intake control: moving beyond palatability. Obes Rev.

[6] Aschenbrenner K (2008). The influence of olfactory loss on dietary behaviors. Laryngoscope.

[7] Birch LL (1999). Development of food preferences. Annu Rev Nutr.

[8] Aliani M (2013). Aroma and taste perceptions with Alzheimer disease and stroke. Crit Rev Food Sci Nutr.

[9] Kremer S, Holthuysen N, Boesveldt S (2014). The influence of olfactory impairment in vital, independently living older persons on their eating behaviour and food liking. Food Qual Prefer.

[10] Boesveldt S (2011). Gustatory and olfactory dysfunction in older adults: a national probability study. Rhinology.

[11] Liu B et al (2019) Relationship between poor olfaction and mortality among community-dwelling older adults: a cohort study. Ann Intern Med 10.7326/M18-0775 PMC7673250 31035288

[12] Rahayel S, Frasnelli J, Joubert S (2012). The effect of Alzheimer's disease and Parkinson's disease on olfaction: a meta-analysis. Behav Brain Res.

[13] Mesholam RI (1998). Olfaction in neurodegenerative disease. Arch Neurol.

[14] Stamps JJ, Bartoshuk LM, Heilman KM (2013). A brief olfactory test for Alzheimer's disease. J Neurol Sci.

[15] Sakai M (2016). Decline of gustatory sensitivity with the progression of Alzheimer's disease. Int Psychogeriatr.

[16] Steinbach S (2010). Taste in mild cognitive impairment and Alzheimer's disease. J Neurol.

[17] Broggio E (2001). Taste impairment in Alzheimer's disease. Revue Neurologique.

[18] Kouzuki M (2018). Comparison of olfactory and gustatory disorders in Alzheimer's disease. Neurol Sci.

[19] Mungas D (1990). Dietary preference for sweet foods in patients with dementia. J Am Geriatr Soc.

[20] Keene JM, Hope T (1997). Hyperphagia in dementia: 2. Food choices and their macronutrient contents in hyperphagia, dementia and ageing. Appetite.

[21] Serby M, Larson P, Kalkstein BA (1991). The nature and course of olfactory deficits in Alzheimer’s disease. Am J Psychiatry.

[22] Folstein M, Folstein S, McHugh P (1975). "Mini-mental state" a practical method for grading the cognitive state of patients for the clinician. J Psychiat Res.

[23] van der Flier WM, Scheltens P (2018). Amsterdam dementia cohort: performing research to optimize care. J Alzheimers Dis.

[24] McKhann GM (2011). The diagnosis of dementia due to Alzheimer's disease: recommendations from the National Institute on Aging-Alzheimer's Association workgroups on diagnostic guidelines for Alzheimer's disease. Alzheimers Dement.

[25] Albert MS (2011). The diagnosis of mild cognitive impairment due to Alzheimer's disease: recommendations from the National Institute on Aging-Alzheimer's Association workgroups on diagnostic guidelines for Alzheimer's disease. Alzheimers Dement.

[26] van der Flier WM (2014). Optimizing patient care and research: the Amsterdam Dementia Cohort. J Alzheimers Dis.

[27] Verhage F (1965). Intelligence and age in a Dutch sample. Hum Dev.

[28] Stanciu I (2014). Olfactory impairment and subjective olfactory complaints independently predict conversion to dementia: a longitudinal, population-based study. J Int Neuropsychol Soc.

[29] Hummel T (1997). 'Sniffin' sticks': Olfactory performance assessed by the combined testing of odor identification, odor discrimination and olfactory threshold. Chem Senses.

[30] Hummel T (2007). Normative data for the "Sniffin' Sticks" including tests of odor identification, odor discrimination, and olfactory thresholds: an upgrade based on a group of more than 3,000 subjects. Eur Arch Otorhinolaryngol.

[31] Mueller CA (2003). Quantitative assessment of gustatory function in a clinical context using impregnated "taste strips". Rhinology.

[32] de Bruijn SEM (2017). The reliability and validity of the Macronutrient and Taste Preference Ranking Task: a new method to measure food preferences. Food Qual Prefer.

[33] Lindeboom J (2002). Visual association test to detect early dementia of the Alzheimer type. J Neurol Neurosurg Psychiatry.

[34] Rey A (1964). L’examen clinique en psychologie [The clinical psychological examination].

[35] Reitan R (1955). The relation of the Trail Making Test to organic brain damage. J Consult Psychol.

[36] Wechsler D (1997). Adult Intelligence Scale—Administration and Scoring Manual.

[37] Stroop R (1935). Studies of interference in serial verbal reactions. J Exp Psychol.

[38] Hughes B (1970). Missile wounds of the brain. A study of psychological deficits. J Neurol Neurosurg Psychiatry.

[39] Dubois B (2000). The FAB a frontal assessment battery at bedside. Neurology.

[40] Benton AL (1967). Differential behavioral effects in frontal lobe disease. Neuropsychologia.

[41] Warrington EK, James M (1991). The visual object and space perception battery.

[42] Teunissen C (2009). A consensus protocol for the standardization of cerebrospinal fluid collection and biobanking. Neurology.

[43] Mulder C (2010). Amyloid-beta(1–42), total tau, and phosphorylated tau as cerebrospinal fluid biomarkers for the diagnosis of Alzheimer disease. Clin Chem.

[44] Tijms BM (2018). Unbiased approach to counteract upward drift in cerebrospinal fluid amyloid-beta 1–42 analysis results. Clin Chem.

[45] Murphy C (2002). Prevalence olfactory impairment in older adults. JAMA.

[46] Peters JM (2003). Olfactory function in mild Cognitive impairment and Alzheimer’s Disease: an investigation using psychophysical and electrophysiological techniques. Am J Psychiatry.

[47] Djordjevic J (2008). Olfaction in patients with mild cognitive impairment and Alzheimer's disease. Neurobiol Aging.

[48] Larsson M (1999). Odor identification in normal aging and ealry Alzheimer's disease: effects of retrieval support. Neuropsychology.

[49] Hedner M (2010). Cognitive factors in odor detection, odor discrimination, and odor identification tasks. J Clin Exp Neuropsychol.

[50] Landis BN (2009). "Taste Strips"—a rapid, lateralized, gustatory bedside identification test based on impregnated filter papers. J Neurol.

[51] Griffioen-Roose S (2011). The effect of within-meal protein content and taste on subsequent food choice and satiety. Br J Nutr.

[52] Griffioen-Roose S (2010). Measuring food reward and the transfer effect of sensory specific satiety. Appetite.

