# Supplementary material for: Olfactory and gustatory functioning and food preferences of patients with Alzheimer’s disease and mild cognitive impairment compared to controls: the NUDAD project
Source: J Neurol. 2019 Oct 8;267(1):144–52. doi: 10.1007/s00415-019-09561-0 (PMC6954901; doi:10.1007/s00415-019-09561-0)
Supplement: Supplementary file 1 — Supplementary file1 (DOCX 13 kb) [file 415_2019_9561_MOESM1_ESM.docx]

**Supplementary table A**. Liking scores of the taste intensity preference per diagnosis group

|  | **Controls** | **MCI** | **AD dementia** | **P-value across groups** |
| --- | --- | --- | --- | --- |
| **Lemonade** |  |  |  |  |
| Lowest concentration | 27.7 ± 3.5 | 23.9 ± 4.6 | 25.5 ± 4.1 | 0.814 |
| 2^nd^ lowest | 35.4 ± 3.8 | 39.7 ± 5.0 | 29.7 ± 4.5 | 0.335 |
| Intermediate | 42.8 ± 4.7 | 48.5 ± 6.1 | 40.2 ± 5.4 | 0.595 |
| 2^nd^ highest | 33.2 ± 4.7 | 50.4 ± 6.2 | 48.6 ± 5.5 | 0.054 |
| Highest concentration | 22.5 ± 5.1 | 45.0 ± 6.7† | 47.7 ± 5.9† | **0.005** |
| **Tomato juice** |  |  |  |  |
| Lowest concentration | 35.7 ± 4.2 | 35.8 ± 5.5 | 31.0 ± 4.9 | 0.743 |
| 2^nd^ lowest | 38.7 ± 4.3 | 35.3 ± 5.7 | 34.8 ± 5.0 | 0.829 |
| Intermediate | 45.8 ± 4.5 | 45.5 ± 5.9 | 37.4 ± 5.2 | 0.454 |
| 2^nd^ highest | 35.3 ± 4.0 | 36.9 ± 5.3 | 32.0 ± 4.7 | 0.779 |
| Highest concentration | 13.7 ± 3.0 | 24.1 ± 3.9 | 20.3 ± 3.4 | 0.111 |

Data in Mean ± SE. Tested using age, gender and education adjusted ANOVA with post-hoc Bonferroni adjusted t-tests.

AD= Alzheimer’s disease; MCI= Mild Cognitive Impairment; † significantly different from controls upon post-hoc
